# 16S rDNA Profiling to Reveal the Influence of Seed-Applied Biostimulants on the Rhizosphere of Young Maize Plants

**DOI:** 10.3390/molecules23061461

**Published:** 2018-06-15

**Authors:** Giovanna Visioli, Anna Maria Sanangelantoni, Teofilo Vamerali, Cristian Dal Cortivo, Massimo Blandino

**Affiliations:** 1 Department of Chemistry, Life Sciences and Environmental Sustainability, University of Parma, Parco Area delle Scienze 11/A, 43124 Parma, Italy; sanan@unipr.it; 2 Department of Agronomy, Food, Natural Resources, Animals and the Environment, University of Padua, Viale dell’ Università 16, 35020 Legnaro-Padua, Italy; teofilo.vamerali@unipd.it (T.V.); cristian.dalcortivo@unipd.it (C.D.C.); 3 Department of Agriculture, Forest and Food Sciences, University of Turin, Largo Paolo Braccini 2, 10095 Grugliasco (TO), Italy; massimo.blandino@unito.it

**Keywords:** biostimulants, *Zea mays*, rhizosphere, soil bacterial biodiversity, sustainable agriculture, 16S rDNA sequencing

## Abstract

In an open field trial on two agricultural soils in NW Italy, the impact of two seed-applied biostimulants on the rhizosphere bacterial community of young maize plants was evaluated. The 16S rDNA profiling was carried out on control and treated plant rhizosphere samples collected at the 4-leaf stage and on bulk soil. In both soils, the rhizospheres were significantly enriched in Proteobacteria, Actinobacteria, and Bacteriodetes, while the abundances of Acidobacteria, Cloroflexi and Gemmatimonadetes decreased compared with bulk soil. Among the culturable bacteria genera that showed an increase by both biostimulants, most are known to be beneficial for nutrient uptake, such as *Opitutus*, *Chryseolinea*, *Terrimonas*, *Rhodovastum*, *Cohnella*, *Pseudoduganella* and the species *Anaeromyxobacter dehalogenans*; others are known to be involved in root growth, such as *Niastella*, *Labrys*, *Chloroflexia* and *Thermomonas*; or in plant defence, such as *Ohtaekwangia*, *Quadrisphaera*, *Turneriella*, and *Actinoallomurus*. Both biostimulants were also found to stimulate gen. *Nannocystis*, a potential biocompetitive agent against aflatoxigenic *Aspergillus* moulds. Under controlled conditions, both biostimulants enhanced the shoot and root biomass at the 4–5 leaf stage. We conclude that the biostimulants do not decrease the biodiversity of the microbial community rhizosphere of young maize plants, but stimulate rare bacterial taxa, some involved in plant growth and pathogen resistance, a result that may have implications in improving crop management.

## 1. Introduction

Plant biostimulants have been defined as “material containing substance(s) and/or microorganisms whose function is to stimulate natural processes, to enhance/benefit nutrient uptake, nutrient efficiency, to increase tolerance to abiotic stresses and crop quality when applied to plants or to the rhizosphere” [1]. Many biostimulants contain simple and complex carbohydrates that, when applied to plants, improve the efficiency of plant metabolism by directly acting as a source of energy for rhizosphere microbial populations or by acting as signal molecules to activate defence reactions [2]. Biostimulants improve macro-nutrient uptake (for example, through stimulation of nitrogen metabolism), increase plant tolerance to abiotic stresses [3,4], and ameliorate crop yield and quality, especially under unfavourable environmental conditions [5,6]. Despite the many beneficial effects on plants, the effective use of biostimulants requires a better understanding of their function and their possible influence on endogenous rhizosphere microbial communities (plant *rhizobiome*) in order to improve safety, sustainability, product quality, and optimize industrial production processes [7].

The plant *rhizobiome* is one of the most complex, diverse, and important microbial consortiums in the biosphere and plays a significant role in plant-soil ecosystem functions by contributing to plant nutrition, hormonal control of plant growth, and disease suppression. The *rhizobiome* contains diverse types of plant-growth-promoting rhizobacteria (PGPR), which have beneficial effects on crop productivity [8,9,10]. Biostimulants based on the mixtures of plant growth promoting bacteria and arbuscular mycorrhizae fungi might represent an interesting tool for increasing crop tolerance to alkalinity and salinity and for helping plants withstand soil pathogen stress. However, the efficacy of bacteria and fungi applied directly to the soil, sprayed on foliar surfaces, or coated on plant seed in open field cultivations could be reduced by competition with the resident soil microbial community [11,12]. Biostimulant formulations without microorganisms have the potential to enhance the endogenous rhizosphere microbial community, selecting beneficial bacteria as PGPR, with positive effects on plant growth. To date, the only study that has investigated the effects of amended soils with biostimulants, based on protein hydrolysates, on the alteration of soil microbial community structure is by Tejada et al. [13]. The authors showed a greater soil microbial activity with faster plant establishment on degraded soils due to biostimulant application.

Compared to the other application methods, seed dressing is the basic and the cheapest way to apply specific molecules to the crop at planting. In this way, the amount of product applied to the seed is modest and the effectiveness of this strategy must be carefully evaluated.

Culture-dependent techniques and low-resolution culture-independent molecular methods have been extensively used to profile microbial communities in soil rhizosphere. In the last years, the introduction of high-throughput Next-Generation Sequencing (NGS) technologies has greatly improved studies of bacterial diversity and the first applications to maize rhizospheres have been reported very recently [14,15,16,17,18,19]. NGS is now considered an essential tool for studying the effects of external inputs on the microbial community colonising the rhizosphere of maize plants and can also be applied for revealing the possible effects of biostimulants on the population of beneficial microorganisms in the rhizosphere.

In this study, 16S rDNA profiling was used to map the composition of the bacterial communities of the maize rhizosphere in the first stages of growth. Maize plants were grown from seeds treated with two biostimulants and from untreated seeds in two sites in north-western Italy with different soil types. A preliminary pot trial in a greenhouse was performed to test the effects of both biostimulants on plant growth. The biostimulants were obtained from fermentation of plant materials of differing compositions without the addition of microorganisms. 

The aim of this work was to verify whether the application of two biostimulants (referred to as Bio1 and Bio2) through seed dressing could impact on the relative abundances of bacterial phyla, families and genera and influence the soil bacterial community, with possible beneficial effects on root and shoot growth. Although there has been some work done to study the effects of soil amendments to rhizosphere bacterial populations [20,21], to the best of our knowledge, no studies have yet been published on mapping by 16S rDNA profiling the possible modifications of the rhizosphere microbial community as a result of applying biostimulants to maize seeds.

## 2. Results

### 2.1. Effects of Biostimulants on Plant Growth

**Figure 1:**
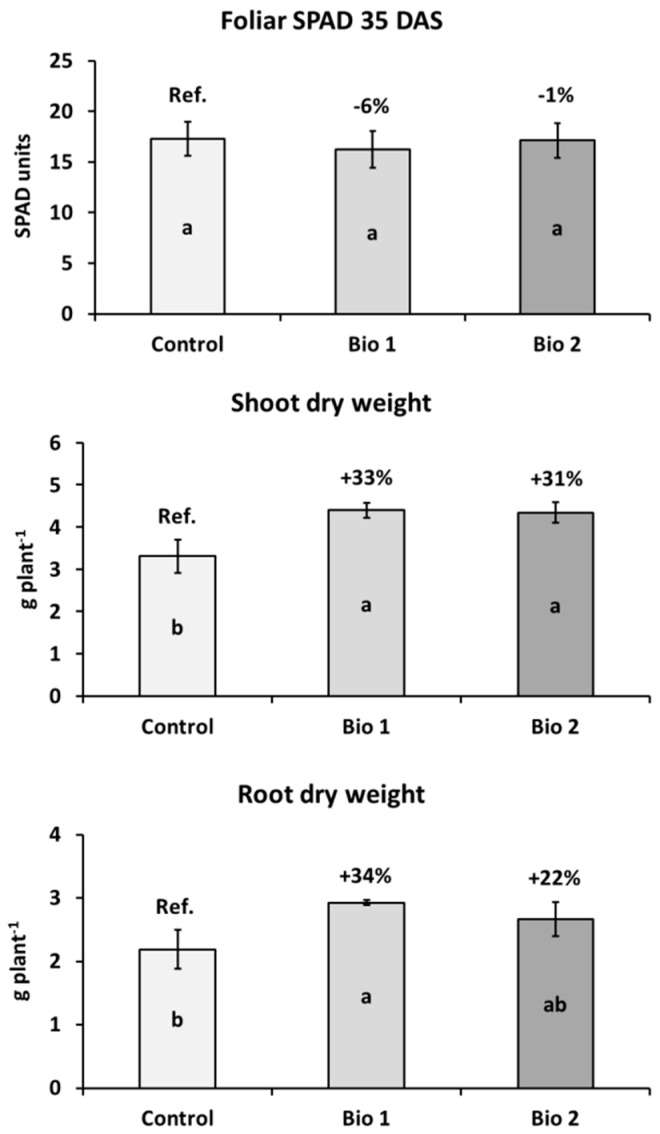
The leaf SPAD values, shoot and root dry biomass (mean ± S.E., n = 3) of pot-cultivated maize (4–5 leaf stage, 35 days after sowing) treated with two seed-coating biostimulants (Bio1 and Bio2) in comparison with untreated controls. Different letters (a,b) indicate significant differences between samples according to the Student Newman-Keuls method (SNK) *(p* ≤ 0.05).

Under controlled conditions (pots in a greenhouse), both biostimulants did not affect the SPAD (Soil Plant Analysis Development) index, as representative of the leaf chlorophyll content, at the 4–5 leaf stage, while enhanced plant growth. Bio1 significantly increased both shoot (+33%) and root (+34%) biomass (*p* ≤ 0.05) as compared to controls, and Bio2 significantly improved shoot weight (+31%) (Figure 1).

### 2.2. Total Bacterial Community: Structure and Diversity

A total of 2,066,787 raw 16S rDNA reads were obtained from 32 soil analyses (four biological replicates, each of BS, RhC, RhBio1, and RhBio2 from the Chivasso and Carignano fields). After filtering and removing the chimaeras, a total of 1,539,514 sequences were retained with mean numbers of total sequences per sample (average read of four replicates) ranging from 47,593 to 60,212 (Table S1).

Bacterial diversity in the BS and in the maize rhizospheres of plants grown from uncoated seeds or from seeds coated with Bio1 and Bio2 at the two sites measured on the basis of Operational Taxonomic Units (OTUs) and calculated bacterial diversity indices, that is, the Shannon diversity index and Chao1 estimator of richness (at 97% sequence similarity) showed there to be no significant differences between the treatments (Table 1).

**Figure 2:**
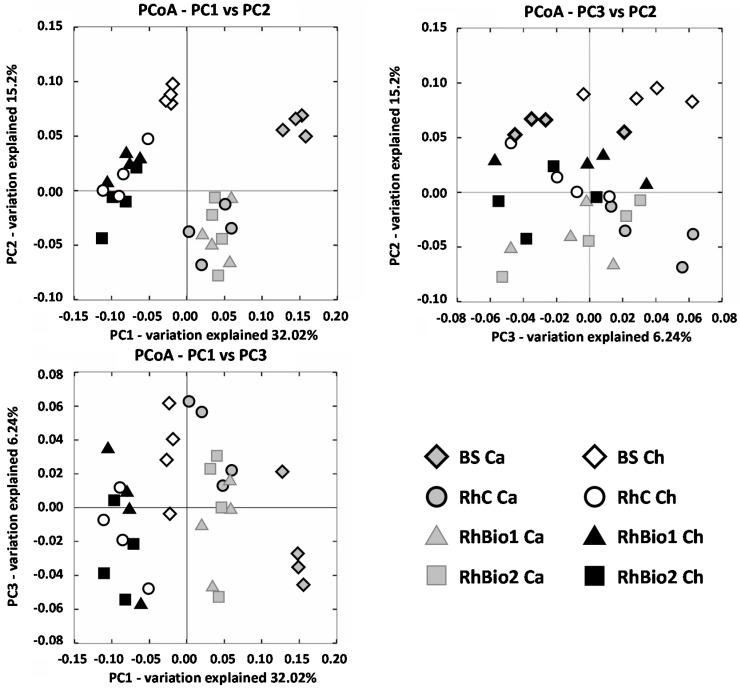
The Principal Coordinate Analysis (PCoA) of the bacterial communities in the different soil samples analysed. Percentages shown along the axes represent the proportions of dissimilarity captured by the axes. Each symbol represents the 16S rRNA gene sequences from each sample, which are depicted in a scale of grays, using a shape for each soil condition (rhombus = bulk soil; circle = control rhizosphere, triangle=rhizosphere with Bio1; square=rhizosphere with Bio2). BS = bulk soil; RhC = rhizosphere of control plants; RhBio1 = rhizosphere of plants from seeds treated with Bio1; RhBio2 = rhizosphere of plants from seeds treated with Bio2; Ca = Carignano site; Ch = Chivasso site.

Principal Coordinate Analysis (PCoA) was carried out to reduce the number of variables in the data while maintaining as much variance as possible. This showed that the four replicates usually clustered close together (Figure 2), demonstrating the reproducibility of these bacterial profiles. 

The rhizosphere and BS bacterial communities were clearly separated from each other (Figure 2), indicating a significant impact of maize roots on selecting and shaping the rhizosphere bacterial community. The effect of the locality/soil type was the main factor in the first principle coordinate axis (PCo1), which accounted for 32.02% of the total variation. Rhizosphere bacterial communities are also separated from their BS, the main factor in the second coordinate axis (PCo2), contributing to 15.20% of the variation. The effect of the biostimulants on the rhizosphere appeared to be somewhat explained by the third coordinate axis (PCo3) and accounted only for a small variability (6.24%).

**Figure 3:**
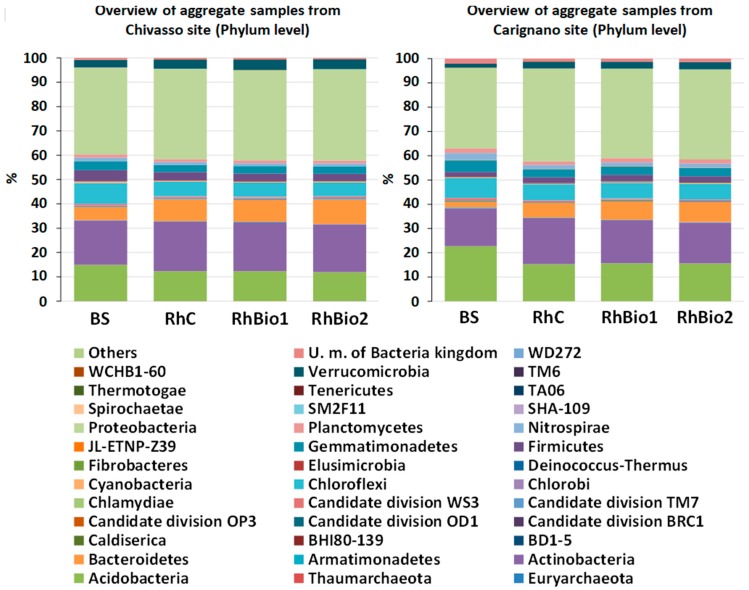
Taxonomic distribution at the phylum level based on 16S rDNA reads across the four sample types from the Carignano site (right) and the Chivasso site (left). BS = bulk soil; RhC = rhizosphere of control plants; RhBio1 = rhizosphere of plants from seeds coated with Bio1; RhBio2 = rhizosphere of plants from seeds coated with Bio2.

All 1,539,514 sequences were assigned to 36 phyla, 21 of which were already classified, while the others belonged to unidentified microbes represented only by sequences in the SILVA database (Figure 3), and 460 families, 78 of which had relative abundances >0.6% and contributed about 80% of the variation (Figures S1 and S2).

A total of 829 genera were also identified, about 50 of which had relative abundances >0.6% and high similarity with known culturable bacteria, while about 50 were attributed to non-culturable bacteria (Figures S3 and S4).

**Figure 4:**
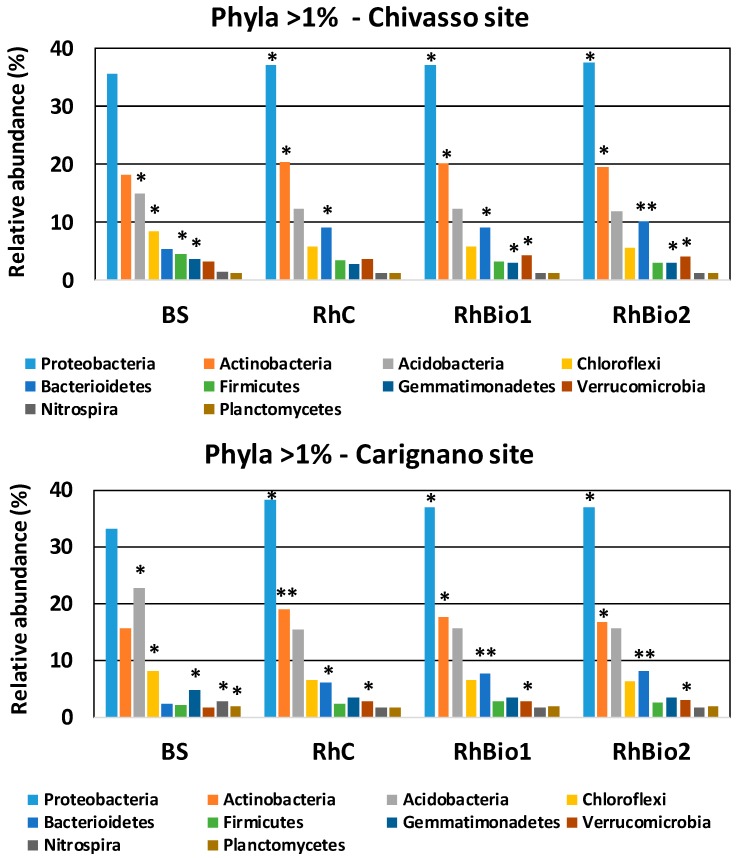
The percentage of phyla present at >1% in all the soil samples from the two sites, Chivasso and Carignano, based on 16S rDNA reads. BS = bulk soil; RhC = rhizosphere of control plants; RhBio1 = rhizosphere of plants from seeds coated with Bio1; RhBio2 = rhizosphere of plants from seeds coated with Bio2. Data from each soil type are derived from four biological replicates (each consisting of the rhizosphere of six plants). Significantly different values among treatments within same site are indicated with asterisks, (* *p* ≤ 0.05; ** *p* ≤ 0.01).

The most abundant of all the phyla present were Proteobacteria, Acidobacteria, and Actinobacteria, together accounting for 68.8% of the total sequences occurring in either BS samples from Chivasso, BS from Carignano, Rh from Chivasso, or Rh from Carignano. Phyla Chloroflexi, Bacteriodetes, Nitrospirae, Firmicutes, Gemmatimonadetes, Planctomycetes, and Verrucomicrobia were less abundant, accounting for 1% to 8% of the total (Figure 4). The rhizospheres were significantly enriched with a subset of phyla at both sites (Figure 4), in particular, Proteobacteria, Actinobacteria, and Bacteriodetes. On the other hand, the abundances of phyla Acidobacteria, Cloroflexi, and Gemmatimonadetes were lower in the rhizospheres from both soils than in the BS. These phyla accounted for an average of 31% of the total bacterial community in BS (27% at the Chivasso site, 36% at Carignano), but only an average of 22% in the rhizospheres (21% at Chivasso, 25% at Carignano). Moreover, a much higher proportion of unclassified sequences were detected in the BS (2% of total) than in the Rh samples (0.02% of total).

A noticeable effect of soil type on bacteria abundance was evident: Acidobacteria was the second most abundant phylum in the BS from Carignano (22.7% of the total), but was only 14.9% of the total in the BS from Chivasso. Actinobacteria, on the other hand, was the second most abundant phylum in the BS from Chivasso (18.2% of total) and the third in the BS from Carignano (15.6% of total). Other phyla which differed between the two soils were Bacterioidetes, which were more abundant in the BS from Chivasso (5.4% of total) than in the BS from Carignano (2.35% of total), and Verrucomicrobia, which accounted for 3.18% of the total in the BS from Chivasso and only 1.56% in the BS from Carignano (Figure 4).

### 2.3. Effects of Biostimulants on the Rhizospheric Microbial Community of Maize

With regard to the effects of biostimulants on the bacterial community, the only phylum showing significant increases were the Bacteroidetes, which were +2.09% in Rh from Carignano, +1.17% in Rh from Chivasso under Bio2, and +1.5% in Rh from Carignano under Bio1, compared with the respective controls (Figure 4).

Taking all the OTUs present in the different rhizosphere soil samples, 49 taxa were identified at Chivasso and 58 at Carignano, with significant variation (*p*-value ≤ 0.05) in RhBio1 and RhBio2 compared with RhC (Table 2 and Table 3). At the Chivasso site, 23 taxa showed significant variation with Bio1, and 31 with Bio2, including five showing significant variation with both biostimulants (Table 2), while in Carignano, 26 taxa exhibited significant variation (*p*-value ≤ 0.05) with Bio1, and 37 with Bio2, including five showing significant variation with both biostimulants (Table 3). Of these, we found significant increases in abundance in only 10 taxa with Bio1 and in 11 taxa with Bio2 at Chivasso, while at Carignano, 13 taxa increased significantly with Bio 1 and 17 with Bio2 (*p*-values ≤ 0.05) (Table 2 and Table 3, Table S2). Only 4 taxa at Chivasso and 4 at Carignano increased significantly with both Bio1 and Bio2 (Table 2 and Table 3, Table S2).

Of these bacteria, the obligate anaerobic *Opitutus* genus belonging to the Verrucomicrobia phylum [22], known for its part in the nitrogen cycle in reducing nitrate to nitrite, was found to increase significantly under both biostimulants at the Chivasso site. Non-culturable bacteria belonging to the PHOS-HE51 family also increased; these are members of the Cytophagales order, known for their ammonia oxidation ability at low dissolved oxygen concentrations [23] (Table 2). Interestingly, the *Nannocystis* genus belonging to the Sorangiaceae family was found to be significantly stimulated at the Chivasso site by both Bio1 and Bio2 (+20% with Bio1, +31% with Bio2 compared with untreated controls). Members of this bacterial genus produce metabolites like siderophores, nannochelins, steroids, germacrane and geosmin, and are potential biocompetitive agents against *Aspergillus flavus* and *Aspergillus parasiticus* [24]. Other bacteria involved in N cycling showing a significant increase with Bio2 and also positively modulated (although not statistically significantly) with Bio1 belonged to the *Terrimonas* and *Rhodovastum* genera. The *Niastella* genus, which is an indole acetic acid (IAA) producer, some non-culturable bacteria of the Solimonadaceae family with antimicrobial activity, and *Chryseolinea* growing on mono- and disaccharides, and organic acids also increased significantly with Bio2 and exhibited positive modulation with Bio1. 

As for the Carignano site, the four bacterial taxa showing significant increases with both Bio1 and Bio2 belong to the Deltaproteobacteria, class, and the *Isosphaera* and the *Actinoallomurus* genera which include phytohormone-producing bacteria with plant-growth-promoting properties, as well as some being identified as *Anaeromyxobacter dehalogenans* and *Planctomycete* LX80, which are bacteria known to be involved in N cycling (Table S2 and references within). Other bacteria of the Chitinophagaceae family, Chloroflexia class, and *Isophaera* genus, which are IAA producers, P solubilisers, and ACC deaminase producers exhibited similar trends with both Bio1 and Bio2, although the increases were significant only with Bio1. Bacteria of genera *Quadrisphaera*, *Ohtaekwangia*, and *Turneriaella* and of the Anaerolinaceae and Thermoactinomycetaceae families, which are known to possess antimicrobial activity, significantly increased in abundance only with Bio2, along with bacteria of *Pseudoduganella*, *Thermomonas* (N-fixing bacteria) and the *Labrys* genera, known as a phytohormone producer. The *Nannocystis* genus was found to be significantly stimulated at the Carignano site only by Bio1 (+25% compared with controls) (Table 3).

Overall, 40% of the bacterial taxa exhibiting significant increases (*p*-value ≤ 0.05) with Bio1 or Bio2 have a putative diazotrophic function and are involved in N-cycling; 33% are known to produce molecules with antimicrobial activity, and 25% are known to produce phytohormones (such as indole-3-acetic acid), have ACC-deaminase activity, and produce siderophores and other substances stimulating plant growth (Table 4).

## 3. Discussion

The application of biostimulant products to the soil, the foliar surface, or directly as a seed coating is considered one of the most promising sustainable agronomic practices for increasing the efficiency of nutrient uptake by plants while avoiding high inputs of chemical fertilisers in widely-cultivated crops such as maize [25,26,27]. Agricultural biostimulants, which may be formulated from different compounds, substances and microorganisms, are applied to achieve improved crop vigour, yield, quality, and tolerance to abiotic stresses [6,28]. Biostimulants have been demonstrated to foster plant growth and development throughout the crop life cycle from seed germination to plant maturity by improving the efficiency of the plant’s metabolism and by increasing the yield and crop quality of various species. Plant tolerance and recovery from abiotic stresses have been demonstrated upon biostimulant application, as well as an improvement in nutrient assimilation, translocation, and use [7].

Under controlled conditions, we evidenced the growth enhancement effect of the two biostimulants at early growth stages of maize at both the shoot and root level, although with some variations among products. Bio1 was more effective than Bio2, particularly at the root level, probably due to auxins and gibberellins, which have a central role in root expansion and shoot development [29,30]. On the other hand, the abundance of carbon and nitrogen of Bio2 may have had a more direct role on the rhizosphere microorganisms in early stages of plant development providing sugars and nitrogen for microbial metabolism and cues for spore germination [31,32]. We did not study soil microbiology of these pot-grown plants, nor did we assess the ability of these biostimulants to affect plant growth in the field, so we can only speculate about a correlation between the biostimulants’ effect on plant growth and their effect on rhizosphere microbiology. We hypothesise that, despite their lack of microbes, these biostimulants could affect the composition and abundance of rhizosphere bacterial communities in different soil types. As far as the belowground biodiversity is concerned, there were evident differences between the bacterial communities of the two bulk soils (BS) of Carignano and Chivasso and between bulk soils and their respective rhizospheres, but we only observed modest influences of the biostimulants on the abundance of some rare rhizobacterial species, suggesting that the amount and/or persistence of the added biostimulants were probably low. Future experiments with higher concentrations of biostimulant might help in clarifying the potential of these compounds to alter the soil bacterial population.

Soil texture and the content of organic matter appear to be the most important factors affecting rhizosphere bacterial diversity in maize [17,33]. Previous studies have also indicated that environmental variability, such as soil pH and moisture content, likely interact to shape the rhizobiome in this crop [34,35]. 

In our work, the total number of genera found in the maize rhizosphere was higher than in previous studies [14,15], and the number of total sequences per sample (Table S1) indicated that the entire biodiversity had probably been covered, with high biodiversity in both bulk soil and rhizosphere. In the literature, the OTUs richness is reported to vary considerably depending on the soil type, agricultural management, species selection, and plant age [36,37]. As far as maize is concerned, the numbers of OTUs for a similar number of sequences were found higher or similar in the bulk soil than in the rhizosphere depending on the soil type [19,38]. Moreover, a significantly higher functional biodiversity was found in the rhizosphere than in the bulk soil, suggesting that a broad diversity of metabolic activities is enhanced in strict contact with plant roots [18]. The small differences in the number of OTUS between BS and rhizosphere can be ascribed to the fact that our soils have been used from intensive maize cultivation for many years.

Proteobacteria and Actinobacteria were found to be the most abundant phyla, more present in the maize rhizosphere samples than in the BS. They were found to be an important part of the core rhizobiome of maize, maintaining various normal functions and possibly controlling soil-borne pathogens [18,19,39]. Proteobacteria, in particular, have been found in the rhizosphere of most of the plant species. This phylum comprises most of the r-selected fast-growing taxa, which fluctuate opportunistically according to different environmental stimuli. In contrast to the rhizosphere, BS is generally considered to be rich in K-selected microbiota with more stable population sizes. In this study, the enriched phyla in the BS included Acidobacteria, Chloroflexi, and Plantomycetes, which have been previously described as soil oligotrophs [40], in agreement with other studies comparing the maize rhizosphere with BS samples [17].

Another interesting result that emerged from this study was that 50% of the bacteria genera with relative abundances >0.6% were non-culturable, revealing the complexity of the maize rhizobiome.

No differences in the most abundant taxa were found between the rhizospheres of maize plants grown from seeds treated with Bio1 and treated with Bio2 compared with the untreated controls. The specific biostimulants we investigated, when added in the small amounts tested here as seed treatments do not affect the existing structure of the microbial community, regardless of soil type, since they did not decrease the overall rhizosphere biodiversity. Only a few low-abundant taxa showed significant changes in abundance in the rhizosphere of plants grown from seeds treated with biostimulant products compared with those grown from untreted seeds (controls). The ones identified included PGPR involved in N-cycling, and those producing antimicrobial molecules or phytohormones. These small changes in bacterial abundance makes it tempting to speculate that biostimulant products may not only have a direct beneficial effect on plant health and root growth by interacting with plant signalling cascades, but they may also have an indirect effect through the stimulation of rhizosphere bacteria to produce molecules of benefit to the plants [7]. An interesting but modest change that we observed was that the *Nannocystis* genus was significantly stimulated in the maize rhizosphere following treatment with one or the other biostimulant in the loam soil of Chivasso, and with Bio1 in the silty-loam of Carignano. This genus belongs to the *Micrococcales*, which are believed to play a major role in controlling the population of many plant diseases caused by bacteria and fungi in aerated soil [41]. Several members of the *Micrococcales* order have been reported to antagonise soil-borne pathogens [42]. The species *Nannocistis exendens*, in particular, has been reported to be a potential biocompetitive agent against aflatoxigenic moulds [24], a key issue in maize globally, requiring further investigation. In conclusion, this study analysed 16S rDNA profiles of rhizosphere bacterial community of maize plants grown from seeds treated with biostimulants with the aim to develop this technique for seed treatment product certification before their use in large-scale agriculture. Our findings showed that the majority of bacterial taxa of the maize rhizosphere are not altered by these two seed-applied biostimulants in two different and representative soil types of NW Italy. These two biostimulants appear to be able to specifically enhance some bacterial taxa with potential to affect the antibiosis against phytopathogenic fungi, suggesting the need of further studies to prove their ability in selectively enhance populations of beneficial microbes in maize rhizospheres.

## 4. Materials and Methods

### 4.1. Biostimulants Description

Two different biostimulants were applied to the seeds of the maize hybrid SY-Hydro (Syngenta, Basel, Switzerland), namely Bio1 at 300 mL in 100 kg seeds (A18639B, under patenting by Syngenta Crop. Protection AG) containing seaweed and fermented vegetable extracts together with phytohormones (auxins and gibberellins), vitamins, and EDTA-chelated Cu, Zn, and Fe (50% DW); and Bio2 at 200 mL in 100 kg seeds (A21814A under patenting by Syngenta Crop. Protection AG) containing 14.2% organic carbon (as molasses), 3.1% N (as aminoacids and urea), 3% potassium oxide, and K, Mo, Mn (75% DW). Neither biostimulant contained biocides or microorganisms. The two biostimulants were compared with the untreated seeds (controls).

### 4.2. Evaluation of the Biostimulants’ Effects on Plant Growth

In order to evaluate the effects of the two biostimulants on plant growth in the first growth stages, a pot trial was carried out in 2016 before performing the field experiment. Plants were grown in cylindrical PVC pots (22 cm high, 20 cm diameter, 6 L volume) containing a mixture of silty-loam soil collected from the Carignano site (see below) and fine sand (1:1 *w*/*w*) to facilitate water drainage and root collection at end of experiment. Before pot filling, the soil was fertilised with 100 kg of N, 150 kg of P_2_O_5_, and 300 kg of K_2_O on a hectare basis. Within a completely randomised experimental design with 4 replicates, one plant per pot was grown for 35 days up to the 4–5 leaf stage, under 15/25 °C, 14/10 h day/night conditions within a greenhouse. Plants were regularly watered during the whole trial period. At harvest, the photosynthetic activity was measured in SPAD (Soil Plant Analysis Development) units in the last fully developed leaf with a hand-held 502-chlorophyll meter (Konica-Minolta, Hong Kong, China). The shoot and root biomasses were revealed after soil washing and oven-drying for 24 h at 105 °C.

### 4.3. Site Description and Soil Sample Collection

A field experiment was carried out during the 2016 growing season in two locations (district of Turin) representing different conditions for maize cultivation in north-western Italy: one at Chivasso with a shallow loam soil, the other at Carignano with a deep silty-loam soil. The main physical and chemical parameters of the soils are reported in Table 5.

In each location, the treated and control seeds were assigned to the experimental plots using a completely randomised block design, with 4 replicates. Each plot measured 30 m^2^ (10 × 3 m) and consisted of 4 rows, 0.75 m apart. The previous crop was maize at both sites. Planting was carried out after autumn ploughing to a depth of 0.3 m incorporating crop residues into the soil, followed by disk harrowing to prepare a proper seedbed. Mechanical planting was carried out on 23 March 2016 in Chivasso and on 30 March 2016 in Carignano. The crop density was approximately 7.5 plants per m^2^. Before soil harrowing, 150 kg ha^−1^ of K_2_O were applied as potassium chloride. No organic fertilisers (livestock manure or slurry) had been applied at either site in the three previous growing seasons. At sowing, no starter fertilisers were applied to the seed furrow.

Maize plants for 16S rDNA profiling were collected from the experimental sites at the 4-leaf growth stage (GS) [43] on 3 May in Chivasso and 13 May in Carignano. Four biological replicates for each treatment were collected in both sites: bulk soil (BS) and rhizosphere of plants grown from control seeds (RhC); rhizosphere of plants grown from seeds coated with Biostimulant 1 (RhBio1), and rhizosphere of plants grown from seeds coated with Biostimulant 2 (RhBio2). Each biological replicate was obtained from each plot by collecting a soil monolith of 0.2 m depth, containing the root system and the surrounding soil of 6 individual plants (technical replicates). Plant collection occurred in the 2 central rows of each plot. Bulk soil, without maize roots, was sampled by digging 0.2-m deep soil monoliths from the inter-row space (devoid of plant roots) of the untreated controls with again 4 replicates at each site. To collect the rhizosphere soil, plants were gently extracted from the ground and after removing most of the soil by shaking the remaining rhizosphere soil adhering to the roots was carefully collected with a small sterile brush. The rhizosphere soil samples from the six plants of each replicate were pooled to obtain >2 g of sample, which was placed in a sterile Falcon tube for later analysis. An overall amount of 32 samples (4 treatments × 4 biological replications × 2 sites) were collected. Samples were immediately stored at −80 °C until DNA extraction.

### 4.4. DNA Extraction from Rhizospheric Soils

DNA was extracted from 0.5 g of Rh and BS soil samples with the FastDNA^®^ Spin Kit for Soil (MP Biomedicals, Santa Ana, CA, USA) according to the manufacturer’s protocols, visualised by electrophoresis on 0.8% (*w*/*v*) agarose gels to test for DNA integrity, and quantified by Nanodrop ND1000 (Thermo Fisher Scientific, Waltham, MA, USA).

### 4.5. Rhizobiome Identification by 16S rRNA Gene Amplification and Sequencing, and Data Analysis

Partial 16S rDNA sequences were amplified from extracted DNA using the primer pair Probio_Uni/Probio_Rev and targeting the V3 region of the 16S rRNA gene sequence [44]. After amplification and amplicon checks, the 16S rDNA was sequenced with a MiSeq (Illumina, San Diego, CA, USA) and analysed at the DNA sequencing facility of GenProbio srl (www.genprobio.com) according to a previously reported protocol [45]. The biodiversity of the samples (alpha-diversity) was calculated with Chao1 and Shannon indices. Similarities between samples (beta-diversity) were calculated by weighted UniFrac [46]. The range of similarities was calculated between the values 0 and 1. PCoA of beta-diversity was performed with QIIME [47]. Differences in relative abundances of taxonomic units between samples were tested by one-way Analysis of Variance (ANOVA). Statistical analyses were performed by IBM SSPS Statistics Software (www.ibim.com/software/it/analytics/spss/). Bacterial taxa with *p*-values ≤ 0.05 were selected and identified as the phylotypes or bacterial groups that were significantly influenced by Bio1 and Bio2, and the two soil types.

The 16S rDNA reads were sent to GenBank to obtain their under-accession numbers. They are available as bioproject PRJNA387557—with the following accession numbers: BS Ch (replicates 1–4): SAMN07169331, SAMN07172411, SAMN07172412, SAMN07172413; RhC Ch (replicates 1–4): SAMN07169332, SAMN07172414, SAMN07172415, SAMN07172416; RhChBio1 (replicates 1–4): SAMN07169333, SAMN07172417, SAMN07172418, SAMN07172419; RhChBio2 (replicates 1–4): SAMN07169334, SAMN07172420, SAMN07172424, SAMN07172425; BS Ca (replicates 1–4): SAMN07169335, SAMN07172426, SAMN07172493, SAMN07172494; RhC Ca (replicates 1–4): SAMN07169336, SAMN07172495, SAMN07172496, SAMN07172529; RhCaBio1 (replicates 1–4): SAMN07169337, SAMN07172497, SAMN07172498, SAMN07172499; RhCaBio2 (replicates 1–4): SAMN07169338, SAMN07172500, SAMN07172501, SAMN07172502.

## Figures and Tables

**Table 1:** The bacterial diversity indices (at 97% sequence similarity) in bulk soils (BS), and the rhizospheres of untreated control maize plants (RhC) and of plants treated with Bio1 (RhBio1) and Bio2 (RhBio2) in two sites.

Sites	Treatment	Number of OTUs	Chao1 Estimate of Richness	Shannon’s Diversity Index
**Carignano**	BS	4973 ± 96a	6135 ± 148a	9.982 ± 0.021a
	RhC	4824 ± 122a	6107 ± 65a	9.813 ± 0.066a
	RhBio1	5035 ± 125a	6114 ± 45a	9.832 ± 0.115a
	RhBio2	4970 ± 258a	6207 ± 123a	9.883 ± 0.104a
**Chivasso**	BS	5522 ± 327a	6397 ± 464a	9.948 ± 0.244a
	RhC	5271 ± 132a	6260 ± 182a	10.005 ± 0.103a
	RhBio1	5203 ± 148a	6242 ± 115a	10.041 ± 0.117a
	RhBio2	5023 ± 622a	6210 ± 130a	10.067 ± 0.108a

Values are means of four replicate 16S rDNA datasets. The same letter (a) indicate no significant differences between samples according to the Student Newman-Keuls method (SNK) *(p* ≤ 0.05).

**Table 2:** The bacterial taxa present at the Chivasso site with at least one significant variation (*p*-value ≤ 0.05) in the rhizospheres of maize plants grown from seeds treated with Bio1 and Bio2 compared with untreated controls. Data reported are presented as the percentages of the abundance of the mean values of 4 biological replicates.

Taxonomy	RhC	RhBio1	Relative Difference ^a^	*p*-Value	RhBio2	Relative Difference ^a^	*p*-Value
*U.M. of Subgroup 5 order*	0.01%	0.004%	143%	0.038 *	0.007%	35%	0.297
*U.M. of Holophagae class*	0.024%	0.033%	−29%	0.289	0.042%	−44%	0.049 *
*Smaragdicoccus*	0.002%	0.002%	−22%	0.717	0.000%	399%	0.008 **
*Quadrisphaera*	0.004%	0.003%	46%	0.512	0.002%	174%	0.043 *
*Frigoribacterium*	0.000%	0.002%	−81%	0.021 *	0.000%	22%	0.895
*Pseudoclavibacter*	0.01%	0.004%	148%	0.003 *	0.008%	19%	0.469
*Microlunatus*	0.378%	0.311%	21%	0.072	0.267%	41%	0.026 *
*Crossiella*	0.005%	0.002%	249%	0.016 *	0.003%	60%	0.301
*Actinocorallia*	0.024%	0.046%	−47%	0.012 *	0.032%	−24%	0.222
*Euzebya*	0.000%	0.002%	absent in RhC	0.024 *	0.001%	absent in RhC	0.356
*Chryseolinea*	0.233%	0.333%	−30%	0.083	0.351%	−34%	0.046 *
*U.M. of Cytophagaceae family*	0.529%	0.825%	−36%	0.047 *	0.807%	−34%	0.191
*U.M. of AKYH767 family*	0.091%	0.136%	−33%	0.021 *	0.1%	−9%	0.584
*Niastella*	0.208%	0.224%	−7%	0.865	0.317%	−34%	0.0 ***
*Terrimonas*	0.284%	0.328%	−13%	0.181	0.355%	−20%	0.027 *
*U.M. of PHOS-HE51 family*	0.057%	0.096%	−41%	0.027 *	0.089%	−36%	0.148
*U.M. of 08D2Z23 family*	0.001%	0.005%	−89%	0.030 *	0.001%	−64%	0.443
*U.M. of Lineage Iia order (Elusimicrobia class)*	0.041%	0.059%	−30%	0.031 *	0.039%	4%	0.747
*U.M. of Fibrobacteraceae family*	0.069%	0.106%	−34%	0.290	0.097%	−29%	0.023 *
*U.M. of Bacillaceae family*	0.08%	0.06%	34%	0.680	0.045%	78%	0.011 *
*Brevibacillus*	0.013%	0.009%	51%	0.184	0.006%	123%	0.025 *
*U.M. of Paenibacillaceae family*	0.045%	0.027%	67%	0.013 *	0.028%	62%	0.019 *
*Lutispora*	0.008%	0.002%	203%	0.035 *	0.006%	34%	0.500
*Blautia*	0.006%	0.003%	94%	0.045 *	0.003%	124%	0.049 *
*Pseudobutyrivibrio*	0.006%	0.001%	293%	0.044 *	0.004%	45%	0.482
*Desulfitobacterium*	0.003%	0.002%	46%	0.385	0.001%	211%	0.046 *
*Thermincola*	0.004%	0.001%	320%	0.036 *	0.003%	67%	0.370
*U.M. of Peptococcaceae family*	0.002%	0.001%	101%	0.459	0.000%	absent in RhBio2	0.044 *
*Ruminococcus*	0.003%	0.003%	15%	0.787	0.001%	562%	0.042 *
*Phascolarctobacterium*	0.002%	0.003%	−38%	0.658	0.0%	399%	0.008 **
*U.M. of OPB54 class*	0.046%	0.028%	67%	0.007 **	0.03%	55%	0.033 *
*Hyphomicrobium*	0.29%	0.254%	14%	0.124	0.226%	28%	0.009 **
*U.M. of JG35-K1-AG5 family*	0.194%	0.183%	6%	0.568	0.153%	27%	0.046 *
*Rhodobium*	0.564%	0.474%	19%	0.095	0.432%	30%	0.015 *
*U.M. of Xanthobacteraceae family*	2.5%	2.4%	3%	0.767	1.9%	26%	0.028 *
*U.M. of Rhizobiales order*	0.78%	0.748%	4%	0.455	0.656%	19%	0.017 *
*Rhodovastum*	0.004%	0.005%	−23%	0.436	0.008%	−52%	0.046 *
*U.M. of I-10 family*	0.051%	0.034%	49%	0.055	0.037%	37%	0.004 **
*Dongia*	0.007%	0.001%	362%	0.048 *	0.002%	226%	0.099
*U.M. of SM2D12 family*	0.003%	0.004%	−6%	0.901	0.009%	−61%	0.023 *
*U.M. of ARKDMS-49 class*	0.000%	0.002%	absent in RhC	0.041 *	0.000%	absent in RhC and RhBio2	
*Rivibacter*	0.006%	0.006%	−3%	0.913	0.003%	128%	0.037 *
*Nannocystis*	0.036%	0.045%	−20%	0.015 *	0.052%	−31%	0.001 ***
*Aquicella*	0.016%	0.007%	138%	0.019 *	0.015%	4%	0.915
*U.M. of Solimonadaceae family*	0.161%	0.233%	−31%	0.149	0.264%	−39%	0.037 *
*Silanimonas*	0.006%	0.002%	298%	0.048 *	0.004%	54%	0.293
*Stenotrophomonas*	0.033%	0.031%	4%	0.923	0.114%	−71%	0.003 **
*U.M. of SC3-20 class*	0.002%	0.004%	−52%	0.302	0.005%	−64%	0.035 *
*Opitutus*	0.359%	0.556%	−35%	0.016 *	0.515%	−30%	0.045 *

U.M. = Unclassified member; RhC = Rhizosphere of control plants; RhBio1 = Rhizosphere of plants treated with Bio1; RhBio2 = Rhizosphere of plants treated with Bio2. ^a^ Relative difference: (RhC-RhBio1) × 100/RhBio1 and (RhC-RhBio2) × 100/RhBio2. Asterisk indicates a significant value: * *p* ≤ 0.05, ** *p* ≤ 0.01, *** *p* ≤ 0.001.

**Table 3:** The bacterial taxa at the Carignano site with significant variations (*p*-value ≤ 0.05) in the rhizosphere of maize plants grown from seeds treated with Biostiumant 1 and Biostimulant 2 compared with untreated controls. Data reported are presented as the percentages of the abundance of the mean values of 4 biological replicates.

Taxonomy	RhC	RhBio1	Relative Difference ^a^	*p*-Value	RhBio2	Relative Difference ^a^	*p*-Value
*U.M. of Subgroup 19 order*	0.001%	0.002%	−43%	0.428	0.004%	−70%	0.049 *
*Uncultured bacterium gp7*	0.009%	0.019%	−54%	0.009 *	0.007%	32%	0.546
*U.M. of uncultured Thermaerobacter*	0.004%	0.001%	304%	0.042 *	0.006%	−37%	0.438
*Geodermatophilus*	0.613%	0.629%	−3%	0.735	0.523%	17%	0.032 *
*Quadrisphaera*	0.005%	0.007%	−31%	0.154	0.009%	−44%	0.011 *
*U.M. of Cellulomonadaceae family*	0.002%	0.001%	140%	0.261	0.0%	absent in RhBio2	0.048 *
*U.M. of Microbacteriaceae family*	0.228%	0.164%	39%	0.031 *	0.152%	49%	0.008 **
*Nocardioides*	0.71%	0.656%	8%	0.446	0.53%	34%	0.006 **
*Actinoallomurus*	0.000%	0.002%	absent in RhC	0.047 *	0.001%	absent in RhC	0.356
*Gaiella*	1.7%	1.3%	18%	0.071	1.3%	23%	0.012 *
*U.M. of FCPU744 family*	0.006%	0.001%	506%	0.023 *	0.004%	58%	0.320
*U.M. of Thermoleophilia class*	0.005%	0.002%	157%	0.028 *	0.002%	193%	0.068
*Prevotella*	0.002%	0.011%	−80%	0.199	0.027%	−92%	0.017 *
*Hymenobacter*	0.012%	0.011%	10%	0.817	0.004%	224%	0.026 *
*Ohtaekwangia*	0.167%	0.158%	6%	0.659	0.22%	−24%	0.039 *
*U.M. of AKYH767 family*	0.014%	0.007%	105%	0.027 *	0.007%	90%	0.020 *
*U.M. of Chitinophagaceae family*	0.466%	0.521%	−11%	0.030 *	0.511%	−9%	0.386
*U.M. of Sphingobacteriaceae family*	0.109%	0.131%	−17%	0.478	0.078%	40%	0.013 *
*U.M. of Anaerolineaceae family*	1.06%	1.1%	−8%	0.263	1.2%	−11%	0.036 *
*U.M. of Chloroflexia class*	0.001%	0.005%	−76%	0.019 *	0.004%	−72%	0.198
*Nitrolancea*	0.006%	0.005%	27%	0.603	0.003%	130%	0.032 *
*U.M. of LineageIIa order*	0.002%	0.007%	−75%	0.012 *	0.006%	−70%	0.023 *
*Anaerobacillus*	0.004%	0.008%	−53%	0.038	0.004%	7%	0.908
*U.M. of Bacillaceae family*	0.021%	0.028%	−23%	0.455	0.031%	−30%	0.018 *
*Brevibacillus*	0.006%	0.002%	175%	0.046 *	0.004%	82%	0.118
*Cohnella*	0.082%	0.114%	−28%	0.021 *	0.102%	−19%	0.361
*U.M. of Thermoactinomycetaceae family*	0.001%	0.001%	−42%	0.632	0.004%	−85%	0.010 **
*U.M. of Clostridiaceae 1 family*	0.015%	0.006%	141%	0.003 **	0.01%	41%	0.066
*Lutispora*	0.002%	0.000%	absent in RhBio1	0.024 *	0.002%	4%	0.943
*U.M. of C47 class*	0.013%	0.018%	−29%	0.032 *	0.016%	−20%	0.512
*U.M. of Phycisphaeraceae family*	0.000%	0.001%	absent in RhC	0.135	0.002%	absent in RhC	0.027 *
*Planctomycete LX80*	0.006%	0.015%	−56%	0.011 *	0.014%	−53%	0.164
*U.M. of Pla3 lineage class*	0.000%	0.002%	absent in RhC	0.003 **	0.004%	absent in RhC	0.179
*Isosphaera*	0.000%	0.002%	absent in RhC	0.043 *	0.002%	absent in RhC	0.146
*Pir4 lineage (Planctomycetaceae family)*	0.142%	0.18%	−21%	0.100	0.17%	−16%	0.043 *
*Bosea*	0.08%	0.06%	35%	0.121	0.052%	55%	0.021 *
*U.M. of Hyphomicrobiaceae family*	0.057%	0.044%	28%	0.034 *	0.031%	81%	0.010 **
*Microvirga*	0.178%	0.167%	6%	0.582	0.137%	30%	0.039 *
*Rhizomicrobium*	0.051%	0.041%	24%	0.535	0.031%	64%	0.032 *
*Labrys*	0.004%	0.005%	−8%	0.862	0.021%	−79%	0.037 *
*U.M. of MNC12 family*	0.03%	0.02%	50%	0.001 ***	0.019%	55%	0.036 *
*U.M. of MND8 family*	0.129%	0.138%	−7%	0.571	0.166%	−23%	0.026 *
*Ottowia*	0.006%	0.011%	−48%	0.105	0.001%	442%	0.023 *
*Pseudorhodoferax*	0.000%	0.0%	absent in RhBio1	0.000	0.001%	absent in RhC	0.024 *
*Noviherbaspirillum*	0.016%	0.009%	77%	0.204	0.003%	359%	0.048 *
*Pseudoduganella*	0.001%	0.0%	28%	0.870	0.003%	−78%	0.033 *
*U.M. of AKYG597 family*	0.000%	0.000%	absent in RhC	0.356	0.002%	absent in RhC	0.000 ***
*U.M. of FFCH16767 family*	0.007%	0.006%	3%	0.931	0.005%	27%	0.012 *
*Nannocystis*	0.029%	0.039%	−26%	0.036 *	0.027%	8%	0.421
*Sorangium*	0.216%	0.159%	36%	0.050 *	0.198%	9%	0.405
*Anaeromyxobacter dehalogenans*	0.008%	0.016%	−53%	0.001 ***	0.016%	−52%	0.042 *
*U.M. of Deltaproteobacteria class*	0.105%	0.125%	−16%	0.045 *	0.127%	−17%	0.057
*U.M. of CHAB-XI-27 family*	0.002%	0.002%	−13%	0.868	0.0%	absent in RhBio2	0.024 *
*Pseudoxanthomonas*	0.066%	0.051%	29%	0.229	0.033%	102%	0.020 *
*Thermomonas*	0.043%	0.049%	−11%	0.492	0.064%	−33%	0.019 *
*Turneriella*	0.003%	0.004%	−28%	0.650	0.008%	−63%	0.034 *
*U.M. of OPB35 soil group class*	0.507%	0.528%	−4%	0.480	0.616%	−18%	0.014 *
*Others*	0.003%	0.009%	−61%	0.002 **	0.006%	−44%	0.292

U.M. = Unclassified member; RhC = Rhizosphere of control plants; RhBio1 = Rhizosphere of plants grown from seeds treated with Bio1; RhBio2 = Rhizosphere of plants grown from seeds treated with Bio2. Relative difference: (RhC-RhBio1) × 100/RhBio1 and (RhC-RhBio2) × 100/RhBio2. Asterisk indicates a significant variation of the relative difference: * *p* ≤ 0.05, ** *p* ≤ 0.01, *** *p* ≤ 0.001.

**Table 4:** The bacteria taxa showing a significant increase (*p*-value ≤ 0.05) with Bio1 and/or Bio2 and their putative functions in the growth and health of maize plants.

Putative Bacterial Functional Groups	Bacteria Taxa	% ^1^
**N-cycling (diazotrophic bacteria)**	*Opitutus* genus; Cytophagaceae family; *Chryseolinea* genus; *Terrimonas* genus; U.M. PHOS-HE51 family; *Rhodovasum* genus; *Cohnella* genus; *Pseudoduganella* genus; *Anaeromyxobacter dehalogenans* species; *Anaerobacillus* genus; *Planctomicetes* phylum	40
**Antimicrobial activity**	*Solimonadaceae* family; *Nannocystis* genus; *Ohtaekwangia* genus; *Quadrisphaera* genus; *Anaerolinaceae* family; *Turneriella* genus; Thermoactinomycetaceae family; *Actinoallomurus* genus	33
**Production of plant growth hormones, micronutrient bioavailability, S-cycling**	*Niastella* genus; *Chitinophagaceae* family; *Labrys* genus; *Chloroflexia* genus; Isophaera family; *Deltaproteobacteria* class; *Thermomonas* genus	25

^1^ Percentages refer to the total bacteria showing a significant increase with Bio1 and/or Bio2.

**Table 5:** The main physical and chemical characteristics of the soils in the field experiments carried out in 2016 in north-western Italy at Chivasso and Carignano.

Parameters †	Measurement	Loam	Silty-Loam
Location		Chivasso	Carignano
Geographical coordinates		45°12′43″ N; 7°55′45″ E	44°53′21″ N; 7°40′56″ E
Altitude	m a.s.l.	183	245
Soil (USDA classification)		Typic Hapludalfs	Typic Udifluvents
Sand (2–0.05 mm)	%	42.9	28.7
Silt (0.05–0.002 mm)	%	49.3	64.6
Clay (<0.002 mm)	%	7.8	6.7
pH		6.7	8.0
Organic matter	%	1.53	1.45
Total Nitrogen	%	0.081	0.110
C/N		11.0	7.7
Cation Exchange Capacity (C.E.C.)	cmolc/kg	10.6	12.2
Exchangeable Potassium	mg/kg	94	49
Available Phosphorus	mg/kg	36	7
Exchangeable Calcium	mg/kg	875	2002
Exchangeable Magnesium	mg/kg	109	46

† Soil samples were taken from a 0–0.3 m soil depth interval using a cylindrical auger (Eijkelkamp Soil & Water, Giesbeek, The Netherlands).

## Supplementary Materials

The following are available online. Figure S1: Overview of aggregate samples from Chivasso at family level, taxa >0.6%; Figure S2 Overview of aggregate samples from Carignano at family level, taxa >0.6% Figure S3: Overview of aggregate samples from Carignano at genus level, taxa >0.6%; Figure S4: Overview of aggregate samples from Chivasso at genus level, taxa >0.6% Table S1: Data filtering report of single sample runs. Table S2 List of significant abundant taxa at least with one Biostimulant in Chivasso and Carignano sites, and microorganism’s characteristics.

## References

[1] EBIC (2012). What Are Biostimulants?. http://www.biostimulants.eu/about/what-are-biostimulants.

[2] Trouvelot S., Héloir M., Poinssot B., Gauthier A., Paris F., Guillier C. (2014). Carbohydrates in plant immunity and plant protection: Roles and potential application as foliar sprays. Front. Plant Sci..

[3] Calvo P., Nelson L., Kloepper J.W. (2014). Agricultural use of biostimulants. Plant Soil.

[4] Rose M.T., Patti A.F., Little K.R., Brown A.L., Jackson W.R., Cavagnaro T.R., Sparks D.L. (2014). A meta-analysis and review of plant-growth response to humic substances: Practical implication for agriculture. Advances in Agronomy.

[5] Colla G., Rouphael Y., Canaguier R., Svecova E., Cardarelli M. (2014). Biostimulant action of a plant-derived protein hydrolysate produced through enzymatic hydrolysis. Front. Plant Sci..

[6] Popko M., Michalak I., Wilk R., Gramza M., Chojnacka K., Henryk Górecki H. (2018). Effect of the New Plant Growth Biostimulants Based on Amino Acids on Yield and Grain Quality of Winter Wheat. Molecules.

[7] Brown P., Saa S. (2015). Biostimulants in agriculture. Front. Plant Sci..

[8] Pérez-Montaño F., Alías-Villegas C., Bellogín R.A., del Cerro P., Espuny M.R., Jiménez-Guerrero I., López-Baena F.J., Ollero F.J., Cubo T. (2014). Plant growth promotion in cereal and leguminous agricultural important plants: From microorganism capacities to crop production. Microbiol. Res..

[9] Timmusk S., Behers L., Muthoni J., Muraya A., Aronsson A.-C. (2017). Perspectives and challenges of microbial application for crop improvement. Front. Plant Sci..

[10] Vejan P., Abdullah R., Khadiran T., Salmah Ismail S., Boyce A.M. (2016). Role of Plant Growth Promoting Rhizobacteria in Agricultural Sustainability—A Review. Molecules.

[11] Colla G., Hoagland L., Ruzzi M., Cardarelli M., Bonini P., Canaguier R., Rouphael Y. (2017). Biostimulant Action of protein hydrolysates: Unraveling their effects on plant physiology and microbiome. Front. Plant Sci..

[12] Dal Cortivo C., Barion G., Visioli G., Mattarozzi M., Mosca G., Vamerali T. (2017). Increased root growth and nitrogen accumulation followed by PGPR inoculation in common wheat: Assessment of plant-microbe interactions by ESEM. Agric. Ecosyst. Environ..

[13] Tejada M., Benitez C., Gomez I., Parrado J. (2011). Use of biostimulants on soil restoration: Effects on soil biochemical properties and microbial community. Appl. Soil Ecol..

[14] Galeote-Correa D., Bedmar E.J., Fernández-González A.J., Fernández-López M., Arone G.J. (2016). Bacterial communities in the rhizosphere of amilaceous maize (*Zea mays* L.) as assessed by pyrosequencing. Front. Plant Sci..

[15] Bakker M., Chaparro J., Manter D., Vivanco J. (2015). Impacts of bulk soil microbial community structure on rhizosphere microbiomes of *Zea mays*. Plant Soil.

[16] Peiffer J.A., Lery R.E. (2013). Exploring the maize rhizosphere microbiome in the field: A glimpse into highly complex system. Commun. Integr. Biol..

[17] Peiffer J.A., Spor A., Koren O., Jin Z., Tringe S.G., Dangl J.L., Buckler E.S., Ley R.E. (2013). Diversity and heritability of the maize rhizosphere microbiome under field conditions. Proc. Natl. Acad. Sci. USA.

[18] Li X., Rui J., Xiong J., Li J., He Z., Zhou J., Yannarell A.C., Mackie R.I. (2014). Functional potential of soil microbial communities in the maize rhizosphere. PLoS ONE.

[19] Li X., Rui J., Mao Y., Yannarell A., Mackie R. (2014). Dynamics of the bacterial community structure in the rhizosphere of a maize cultivar. Soil Biol. Biochem..

[20] Carrera L.M., Buyer J.S., Vinyard B., Abdul-Baki A.A., Sikora L.J., Teasdale J.R. (2007). Effects of cover crops, compost, and manure amendments on soil microbial community structure in tomato production systems. Appl. Soil Ecol..

[21] Jack A.L.H., Rangarajan A., Culman S.W., Sooksa-Nguan T., Thies J.E. (2011). Choice of organic amendments in tomato transplants has lasting effects on bacterial rhizosphere communities and crop performance in the field. Appl. Soil Ecol..

[22] Van Passel M.W.J., Kant R., Palva A., Copeland A., Lucas S., Lapidus A., Glavina del Rio T., Pitluck S., Goltsman E., Clum A. (2011). Genome sequence of the Verrucomicrobium *Opitutus terrae* PB90-1, an abundant inhabitant of rice paddy soil ecosystems. J. Bacteriol..

[23] Dabert P., Sialve B., Delgenès J.P., Moletta R., Godon J.-J. (2001). Characterisation of the microbial 16S rDNA diversity of an aerobic phosphorus-removal ecosystem and monitoring of its transition to nitrate respiration. Appl. Microbiol. Biotechnol..

[24] Taylor W.J., Draughon F.A. (2001). *Nannocystis exedens*: A potential biocompetitive agent against *Aspergillus flavus* and *Aspergillus parasiticus*. J. Food Prot..

[25] Ertani A., Cavani L., Pizzeghello D., Brandellero E., Altissimo A., Ciavatta C., Nardi S. (2009). Biostimulant activity of two protein hydrolyzates in the growth and nitrogen metabolism of maize seedlings. J. Plant Nutr. Soil Sci..

[26] Ertani A., Schiavon M., Muscolo A., Nardi S. (2013). Alfalfa plant-derived biostimulant stimulate short-term growth of salt stressed Zea mays L.. plants. Plant Soil.

[27] Francis P.B., Larry D., Earnest L.D., Bryant K. (2016). Maize growth and yield response to a biostimulant amendment. J. Crop Improv..

[28] Du Jardin P. (2015). Plant biostimulants: Definition, concept, main categories and regulation. Sci. Hortic..

[29] Fleet C.M., Sun T.P. (2005). A DELLAcate balance: The role of gibberellin in plant morphogenesis. Curr. Opin. Plant Biol..

[30] Overvoorde P., Fukaki H., Beeckman T. (2010). Auxin control of root development. Cold Spring Harb. Perspect. Biol..

[31] Chen S., Subler S., Edwards C.A. (2002). Effects of agricultural biostimulants on soil microbial activity and nitrogen dynamics. Appl. Ecol..

[32] Owen A.G., Jones D.L. (2001). Competition for amino acids between wheat roots and rhizosphere microorganisms and the role of amino acids in plant *N* acquisition. Soil Biol. Biochem..

[33] Castellanos T., Dohrmann A.B., Imfeld G., Baumgarte S., Tebbe C.C. (2009). Search of environmental descriptors to exaplain the variability of the bacterial diversity from maize rhizospheres across regional scale. Eur. J. Soil Biol..

[34] Berg G., Smalla K. (2009). Plant species and soil type cooperatively shape the structure and function of microbial communities in the rhizosphere. FEMS Microbiol. Ecol..

[35] Green J., Bohannan B.J. (2006). Spatial scaling of microbial diversity. Trends Ecol. Evol..

[36] Qiao Q., Wang F., Zhang J., Chen Y., Zhang C., Liu G., Zhang H., Ma C., Zhang J. (2017). The Variation in the Rhizosphere Microbiome of Cotton with Soil Type, Genotype and Developmental Stage. Sci. Rep..

[37] Kamutando C.N., Vikram S., Kamgan-Nkuekam G., Makhalanyane T.P., Greve M., Le Roux J.J., Richardson D.M., Cowan D., Valverde A. (2017). Soil nutritional status and biogeography influence rhizosphere microbial communities associated with the invasive tree *Acacia dealbata*. Sci. Rep..

[38] García-Salamanca A., Molina-Henares M.A., van Dillewijn P., Solano J., Pizarro- Tobías P., Roca A., Duque E., Ramos J.L. (2013). Bacterial diversity in the rhizosphere of maize and the surrounding carbonate-rich bulk soil. Microb. Biotechnol..

[39] Johnston-Monje D., Lundberg D.S., Lazarovits G., Reis V.M., Raizada M.N. (2016). Bacterial populations in juvenile maize rhizosphere originate from both seed and soil. Plant Soil.

[40] Fierer N., Bradford M.A., Jackson R.B. (2007). Toward an ecological classification of soil bacteria. Ecology.

[41] Hocking D., Cook E.D. (1972). Myxobacteria exert partial control of damping-off and root rot disease in container grown tree seedling. Can. J. Microbiol..

[42] Rosenberg E., Varon M., Rosenberg E. (1984). Antibiotics and lytic enzymes. Myxobactera: Development and Cell Interactions.

[43] Lancashire P.D., Bleiholder H., Van Den Boom T., Langeluddeke P., Stauss R., Weber E., Witzenberger A. (1991). A uniform decimal code for growth stages of crops and weeds. Ann. Appl. Biol..

[44] Milani C., Hevia A., Foroni E., Duranti S., Turroni F., Lugli G.A., Sanchez B., Martín R., Gueimonde M., van Sinderen D. (2013). Assessing the fecal microbiota: An optimized ion torrent 16S rRNA gene-based analysis protocol. PLoS ONE.

[45] Mancabelli L., Ferrario C., Milani C., Mangifesta M., Turroni F., Duranti S., Lugli G.A., Viappiani A., Ossiprandi M.C., van Sinderen D. (2016). Insides into the biodiversity of the gut microbiota of broiler chickens. Environ. Microbiol..

[46] Lozupone C., Knight R. (2005). UniFrac: A new phylogenetic method for comparing microbial communities. Appl. Environ. Microbiol..

[47] Caporaso J.G., Kuczynski J., Stombaugh J., Bittinger K., Bushman F.D., Costello E.K., Fierer N., Gonzalez Peña A., Goodrich J.K., Gordon J.I. (2010). QIIME allows analysis of high-throughput community sequencing data. Nat. Meth..

