# Supplementary material for: 16S rDNA Profiling to Reveal the Influence of Seed-Applied Biostimulants on the Rhizosphere of Young Maize Plants
Source: Molecules. 2018 Jun 15;23(6):1461. doi: 10.3390/molecules23061461 (PMC6100521; doi:10.3390/molecules23061461)
Supplement: Supplementary file 1 [file molecules-23-01461-s001.pdf]

Figure S1. Overview of aggregate samples from Chivasso at family level, taxa > 0.6% are shown.

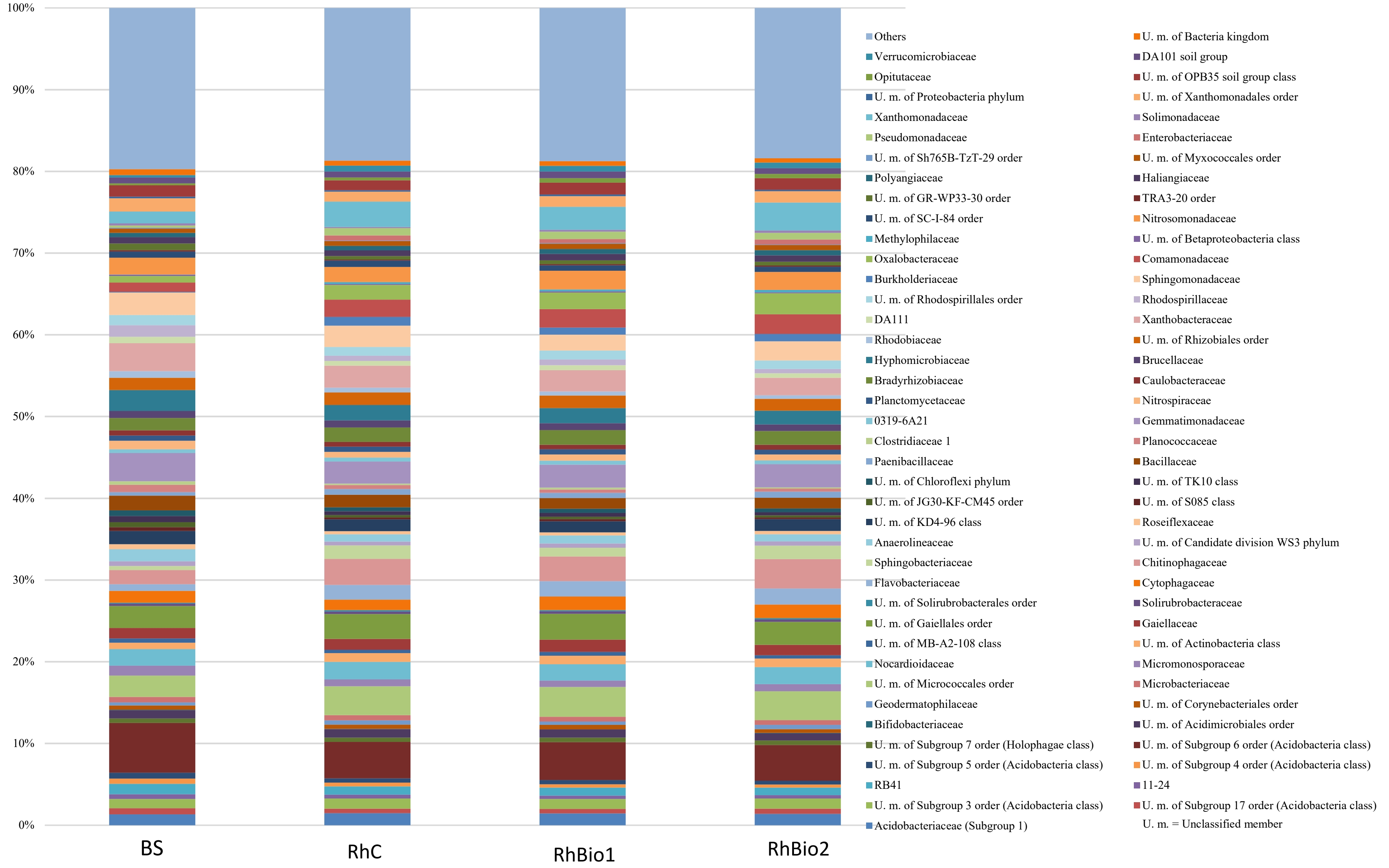

Figure S2. Overview of aggregate samples from Carignano at family level, taxa > 0.6% are shown.

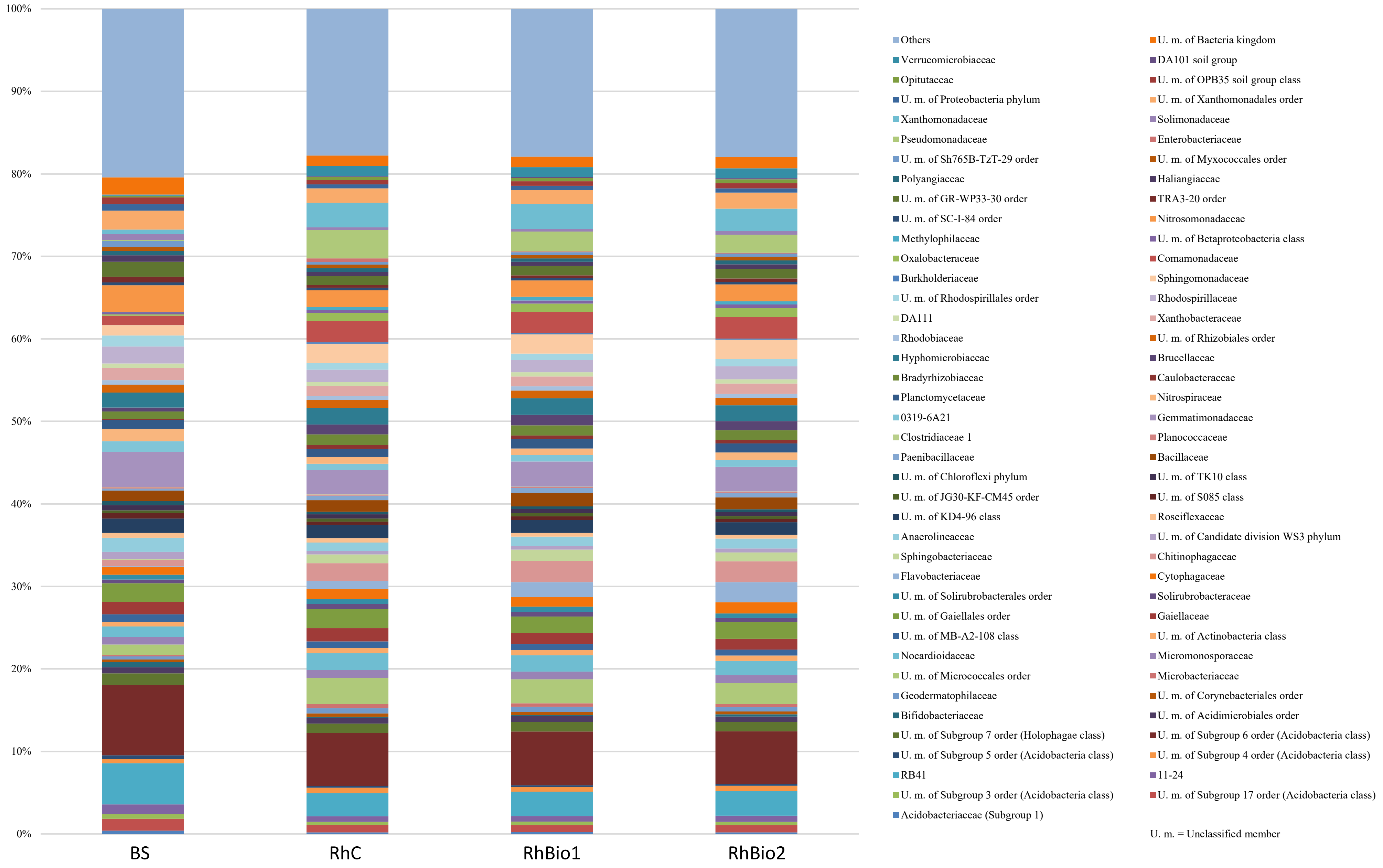

Figure S3. Overview of aggregate samples from Carignano at genus level, taxa>0.6% are shown.

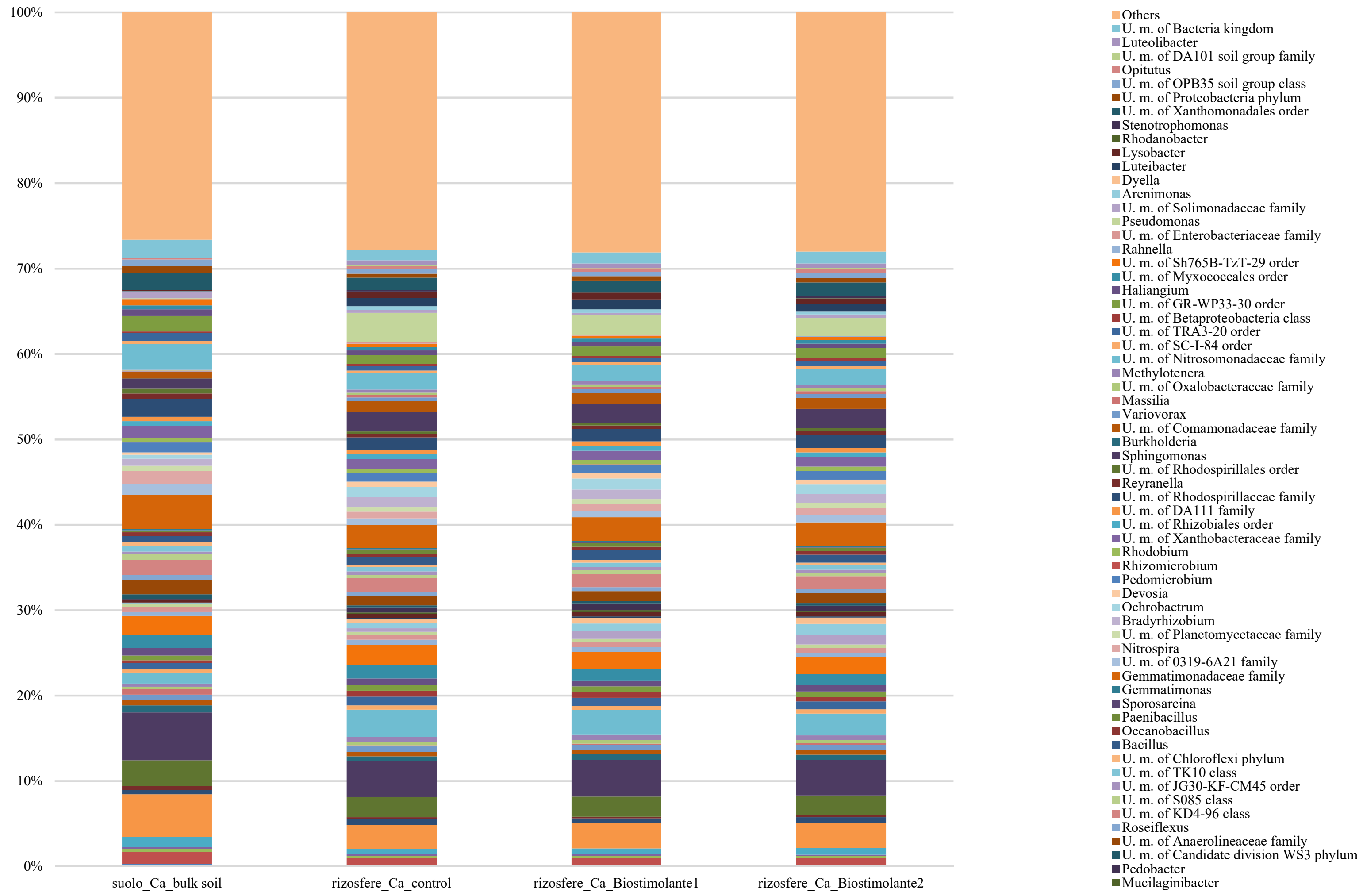

Figure S4. Overview of aggregate samples from Chivasso at genus level, taxa>0.6% are shown.

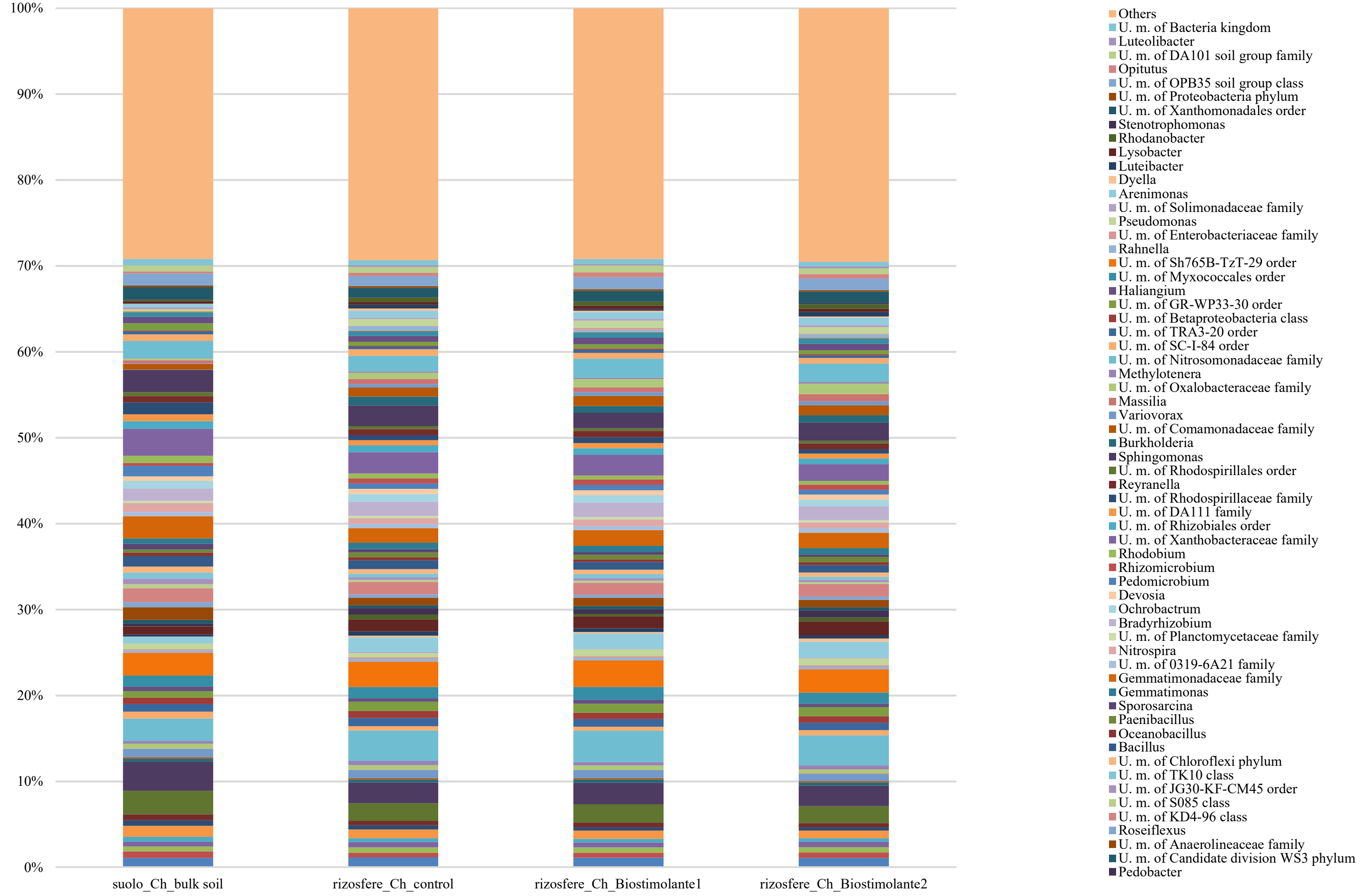

Table S1: Data filtering report of single sample runs. BS= bulk soil; Rh= rhizosphere; Ch=Chivasso site; Ca= Carignano site. Bio1=Biostimulant 1; Bio2= Biostimulant 2. Four biological samples were analysed for each treatment (named 1,2,3,4).

Sample	Number of sequenced pe reads	Number of pe reads with mean quality > 20	Number of merged pe reads	Human sequences	Length outside bounds of 100 and 400	Ambiguous bases	Homopolymers > 7	Mismatch in primers >1	Reverse primer not found	Final Read Number
**BSCh1**	75285	71888	68837	89	0	0	42	1607	66	67033
**BSCh2**	72336	71687	68856	13	0	0	39	2268	40	66496
**BSCh3**	66712	66052	64378	49	0	0	51	1998	52	62228
**BSCh4**	71673	70036	67176	66	0	0	48	3051	57	63954
**RhCh1**	61155	59316	57760	288	0	0	18	2292	51	55111
**RhCh2**	72638	70648	67787	1064	0	0	19	1705	53	64946
**RhCh3**	67095	65028	62731	398	0	0	18	1518	43	60754
**RhCh4**	66256	63213	60119	457	0	0	35	2245	45	57337
**RhChBio1**	68889	66372	64080	1595	0	0	31	1593	47	60814
**RhChBio1**	62286	60762	58872	477	0	0	20	2471	30	55874
**RhChBio1**	60056	58018	56758	161	0	0	33	1675	45	54844
**RhChBio1**	67764	64841	62504	196	0	0	37	2874	44	59351
**RhChBio2**	85535	81384	79529	1671	0	0	36	3145	58	74618
**RhChBio2**	63430	61830	59686	914	0	0	31	1652	43	57046
**RhChBio2**	58036	57224	55568	666	0	0	14	1441	37	53410
**RhChBio2**	41970	41631	39152	157	0	0	14	3555	99	35327
**BSCa1**	65185	62840	60210	46	0	0	89	1568	57	58450
**BSCa2**	57969	54958	52978	33	0	0	82	2229	34	50600
**BSCa3**	62278	59565	57743	65	0	0	90	1889	61	55638
**BSCa4**	59343	56737	54447	30	0	0	70	2924	51	51372
**RhCa1**	60645	59074	57398	393	0	0	85	2400	76	54443
**RhCa2**	60684	57910	55674	1468	0	0	72	1544	50	52540
**RhCa3**	58986	56407	54322	444	0	0	47	1453	71	52307
**RhCa4**	58374	56179	53672	243	0	0	46	2226	38	51119
**RhCaBio1**	65959	63107	60687	901	0	0	69	1512	38	58167
**RhCaBio1**	66246	64156	61822	595	0	0	71	2403	33	58720
**RhCaBio1**	65039	62675	61074	1024	0	0	82	1806	40	58122
**RhCaBio1**	76430	73249	70431	1341	0	0	98	3102	50	65840
**RhCaBio2**	70735	68722	66978	827	0	0	56	2633	45	63417
**RhCaBio2**	60817	59449	57041	392	0	0	50	1564	44	54991
**RhCaBio2**	65632	63467	61367	501	0	0	62	1490	42	59271
**RhCaBio2**	51349	50539	48135	385	0	0	55	2292	29	45374

Table S2. List of significant abundant taxa at least with one Biostimulant in Chivasso and Carignano sites, and microorganisms characteristics.

CHIVASSO SITE

Taxa identified	**Characteristics**	**Refences**
Opitutus genus	N-cycling	Van Passel et al., 2011
Nannocystis genus	Antimicrobial activity and siderophore production	Taylor et al 2001
U.m. PHOS-HE51 family	N-cycling	Dabert et al., 2001
Cytophagaceae family	N cycling	McBride et al., 2014
Chryseolinea genus	N-cycling	Kim et al., 2013
Niastella genus	IAA production, P solubilisation	Chen et al., 2016
Terrimonas genus	N cycling	Jin et al.,2103
Solimonadaceae family	Antimicrobial activity	Zhou et al.,2014
Rhodovastum	N cycling	Okamura et al., 2009

CARIGNANO SITE

Taxa identified	**Characteristics**	**References**
Anaeromyxobacter dehalogenans	N cycling	Sanford et al., 2002
Deltaproteobacteria	Sulfur-reducing bacteria. Plant growth promoting bacteria	Spain et al., 2009
**Planctomycete LX80**	N cycling	Uniprot

Actinoallomurus	PGPR, Phytohormon production	Tang et al., 2013
***Chitinophagaceae* family**	PGPR, IAA ACCdeamynase activity, P solubilisers	Rosenberg, 2014
Chloroflexia	PGPR, Phytohormon production	Pepe-Rnney et al., 2016
Anaerobacillus	Diazotrophic N cycling	Borsodi et al., 2015
Cohnella	Endophyte, N cycling	Garcìa-Fraile et al., 2008
Isosphaera	Phytohormon production	Wang et al., 2002
Nannocystis	Antimicrobial activity	Taylor et al 2001
Quadrisphaera	Antimicrobial activity	Maszenan et al., 2005
Ohtaekwangia	Antimicrobial activity	Correa-Galeote et al., 2016
***Anaerolinaceae* family**	Antimicrobial activity	Nguyen et al., 2016
***Thermoactinomicetaceae* family**	Antimicrobial activity	
Labrys	PGPR. Phytohormon production	Carvalho et al., 2008
Thermomonas	N cycling	Wang et al., 2014
Turneriella	Antimicrobial activity	O’Mahony et al., 2015
Pseudoduganella	N-cycling	Madhaiyan et al., 2013

Borsodi, A.K., Bárány, Á., Krett, G., Márialigeti, K., Szili-Kovács, T., 2015. Diversity and ecological tolerance of bacteria isolated from the rhizosphere of halophyton plants living nearby Kiskunság soda ponds, Hungary. Acta Microbiologica and Immunologica Hungarica. 62:183-97. doi: 10.1556/030.62.2015.2.8.

Carvalho, M.F., De Marco, P., Duque, A.F., Pacheco, C.C., Janssen, D.B., Castro, PM., 2008. *Labrys portucalensis* sp. nov., a fluorobenzene-degrading bacterium isolated from an industrially contaminated sediment in northern Portugal. International Journal of Systematic and Evolutionary Microbiology, 58:692-698. doi: 10.1099/ijs.0.65472-0.

Chen, L., Wang, D., Yang, S., Wang, G., 2016. *Niastella vici* sp. nov., isolated from farmland soil. International Journal of Systematic and Evolutionary Microbiology, 66:1768-72. doi: 10.1099/ijsem.0.000943.

Correa-Galeote, D., Eulogio, J., Bedmar, E.J., Fernández-González, A.J., Fernández-López, M., Gregorio, J., Arone, G.J., 2016 Bacterial communities in the rhizosphere of amilaceous maize (*Zea mays* L.) as assessed by pyrosequencing. Frontiers in Plant Science 7: 1016.

Dabert, P., Sialve, B., Delgenès, JP. Moletta R., Godon J-J 2001. Characterisation of the microbial 16S rDNA diversity of an aerobic phosphorus-removal ecosystem and monitoring of its transition to nitrate respiration Applied Microbiology and Biotechnology 55: 500. doi:10.1007/s002530000529

García-Fraile, P., Velázquez, E., Mateos, P.F., Martínez-Molina, E., Rivas, R., 2008. *Cohnella phaseoli* sp. nov., isolated from root nodules of *Phaseolus coccineus* in Spain, and emended description of the genus *Cohnella*. International Journal of Systematic and Evolutionary Microbiology 58:1855-9. doi: 10.1099/ijs.0.65468-0.

Jin, D., Wang, P., Bai, Z., Jin, B., Yu, Z., Wang, X., Zhuang, G., Zhang, H., 2013. *Terrimonas pekingensis* sp. nov., isolated from bulking sludge, and emended descriptions of the genus *Terrimonas, Terrimonas ferruginea, Terrimonas lutea and Terrimonas aquatica*. International Journal of Systematic and Evolutionary Microbiology 63:1658-64. doi: 10.1099/ijs.0.036848-0.

Kim, J.J., Alkawally, M., Brady, A.L., Rijpstra, W.I., Sinninghe Damsté, J.S., Dunfield, P.F., 2013. *Chryseolinea serpens* gen. nov., sp. nov., a member of the phylum *Bacteroidetes* isolated from soil. International Journal of Systematic and Evolutionary Microbiology 63:654-60. doi: 10.1099/ijs.0.039404-0.
Madhaiyan, M., Poonguzhali ,S., Saravanan, V.S., Hari, K., Lee, K.C., Lee, J.S., 2013. *Duganella sacchari* sp. nov. and *Duganella radicis* sp. nov., two novel species isolated from rhizosphere of field-grown sugar cane. International Journal of Systematic and Evolutionary Microbiology 63:1126-31.

Maszenan, A.M., Tay, J-H., Schumann, P., Jiang, H-L., Tay, T-L. S., 2005 *Quadrisphaera granulorum* gen. nov., sp. nov., a Gram-positive polyphosphate-accumulating coccus in tetrads or aggregates isolated from aerobic granules International Journal of Systematic and Evolutionary Microbiology 55, 1771–1777

Nguyen, N.L., Kim, Y.J., Hoang, V.A., Subramaniyam, S., Kang, J.P., Kang, C.H., Yang, D.C., 2016. Bacterial diversity and community structure in Korean ginseng field soil are shifted by cultivation time. PLoS One.11(5):e0155055. doi: 10.1371/journal.pone.0155055.

O'Mahony, M.M., Henneberger, R., Selvin, J., Kennedy, J., Doohan, F., Julian, R., Marchesi, J.R., Dobson, A.D.W., 2015. Inhibition of the growth of *Bacillus subtilis* DSM10 by a newly discovered antibacterial protein from the soil metagenome Bioengineered. 6: 89–98.

Okamura, K., Hisada, T., Kanbe, T., Hiraishi, A., 2009. *Rhodovastum atsumiense* gen. nov., sp. nov., a phototrophic alphaproteobacterium isolated from paddy soil. Journal of Genetic and Applied Microbiology.55:43-50.

Sanford, R.A., Cole, J.R., Tiedje, J.M., 2002. Characterization and description of *Anaeromyxobacter dehalogenans* gen. nov., sp. nov., an aryl-halorespiring facultative anaerobic myxobacterium. Applied and Environmental Microbiology,. 68:893-900.

Spain, A.M., Krumholz, L.R., Elshahed, M.S., 2009. Abundance, composition, diversity and novelty of soil *Proteobacteria* The ISME Journal 3, 992–1000; doi:10.1038/ismej.2009.43

Tang, Y.L., Lin, H.P., Xie, Q.Y., Li L., Peng, F., Deng, Z. Hong, K., 2013. *Actinoallomurus acanthiterrae* sp. nov., an actinomycete isolated from rhizosphere soil of the mangrove plant *Acanthus ilicifolius*. International Journal of Systematic and Evolutionary Microbiology 63, 1874-1879.

Wang D.I., Wang, L., Wang, G., 2014. *Thermomonas carbonis* sp. nov., isolated from the soil of a coal mine International Journal of Systematic and Evolutionary Microbiology 64, 3631–3635

Wang J, Jenkins C, Webb RI, Fuerst JA 2002. Isolation of *Gemmata*-Like and *Isosphaera*-Like Planctomycete bacteria from soil and freshwater. Applied and Environmental Microbiology, 68: 417–422.
